# Amalgam und Alternativen – Diskussionen zur Quecksilberreduktion in der Umwelt

**DOI:** 10.1007/s00103-021-03355-4

**Published:** 2021-06-18

**Authors:** Roland Frankenberger, Julia Winter, Gottfried Schmalz

**Affiliations:** 1 Abteilung für Zahnerhaltungskunde, Med. Zentrum für Zahn‑, Mund- und Kieferheilkunde, Philipps-Universität Marburg und Universitätsklinikum Gießen und Marburg, Georg-Voigt-Str. 3, 35039 Marburg, Deutschland; 2 Poliklinik für Zahnerhaltung und Parodontologie, Universitätsklinikum Regensburg, Regensburg, Deutschland; 3 Klinik für Parodontologie, ZMK-Kliniken, Universität Bern, Bern, Schweiz

**Keywords:** Karies, Amalgam, Basisversorgung, Quecksilber, Toxikologie, Ästhetik, Füllungstherapie, Umwelt, Caries, Amalgam, Basic care, Mercury, Toxicology, Esthetics, Restorative therapy, Environment

## Abstract

Dentales Amalgam wird seit über 180 Jahren erfolgreich in der zahnärztlichen Füllungstherapie kariöser Läsionen eingesetzt. Es ist langlebig, in der Verarbeitung wenig techniksensitiv und damit fehlertolerant. Seit vielen Jahren befindet sich das dentale Amalgam jedoch in der öffentlichen Diskussion, v. a. wegen seines Quecksilberanteils von ca. 50 %. Seit Veröffentlichung des „Minamata-Übereinkommens“ im Jahr 2013 mit dem primären Ziel, die Ausleitung anthropogenen Quecksilbers in die Umwelt zu reduzieren, ist die zwischenzeitlich fast verstummte Amalgamkritik wieder deutlich lauter geworden. Ein weiterer nicht unerheblicher Nachteil des Amalgams ist die silbrig-schwärzliche Farbe, die heute den ästhetischen Erfordernissen der Patienten oft nicht mehr entspricht.

Der vorliegende Beitrag skizziert die Grundproblematik von Amalgam vor dem Hintergrund multipler biologischer, klinischer und gesundheitspolitischer Aspekte. Es wird Bezug genommen zu möglichen Auswirkungen, insbesondere des Minamata-Übereinkommens, auf gesetzliche Regelungen sowie auf die Verwendung von Werkstoffen und damit auch auf das Versorgungssystem der Zukunft. Außerdem werden mögliche Materialalternativen sowie biomedizinischer Forschungsbedarf auf dem Gebiet der restaurativen Zahnerhaltung dargestellt und abschließend die Frage gestellt, ob wir überhaupt die richtige Diskussion führen.

## Abstract (en)

Dental amalgam has been successfully used for the restoration of carious lesions for more than 180 years. It is clinically characterized by high longevity and low technique sensitivity. For decades, dental amalgam has been discussed in the public, especially due to its roughly 50% mercury content. Since the Minamata Convention was published in 2013 with the primary goal of reducing the anthropogenic mercury release into the environment, the previously muted amalgam discussion has received fresh impetus. Another considerable disadvantage of amalgam is its silver/greyish color, which simply no longer matches patients’ esthetic demands.

The present paper describes the basic problems with amalgam against the background of multiple biological, clinical, and health policy factors. Possible consequences of the Minamata Convention concerning legal regulations as well as the use of dental biomaterials and therefore also relating to the future national healthcare system are discussed. Finally, possible amalgam alternatives and the urgent need for biomedical research towards restorative dentistry are presented, embedded into the crucial question of whether we are actually conducting the correct debate.

## Einleitung

Amalgam ist die Mischung aus einem Legierungspulver („Feilung“, bestehend aus Silber, Zinn und Kupfer) mit Quecksilber (chemisch: Hg). Es wird im plastischen Zustand in die exkavierte und vorbereitete Zahnkavität (Hohlraum) eingebracht und erhärtet schließlich im Mund. Amalgam wird als Füllungsmaterial seit über 180 Jahren verwendet, wobei Zusammensetzung und Verarbeitung zwischenzeitlich grundlegend weiterentwickelt wurden. Noch heute ist Amalgam das Füllungsmaterial, das ohne Zuzahlung bei sozialversicherten Patienten in Deutschland verwendet wird [1]. Das liegt daran, dass es kostengünstig, relativ einfach in der Verarbeitung und robust gegen Kontamination ist. Daher gilt Amalgam noch immer als das klassische Material für die zahnärztliche Basisversorgung im Sinne der Attribute „Wirtschaftlichkeit/Zweckmäßigkeit/Dauerhaftigkeit“ [2].

Trotzdem bestehen aktuell nur 5,3 % der in Deutschland jährlich gelegten Füllungen aus Amalgam [3]. Das liegt daran, dass die Mehrzahl der Patienten eine private Zuzahlung in Kauf nimmt, um aufwendiger zu verarbeitende und vor allem weit weniger fehlertolerante Adhäsivrestaurationen (d. h. geklebte zahnfarbene Füllungen, i. d. R. aus Kompositkunststoffen) zu erhalten. Dieses sog. *minimal-invasive Vorgehen*, d. h. ohne Präparation im Gesunden zur Retentionsgewinnung (Verankerung/Befestigung), ist bei Kompositkunststoffen insbesondere bei der Erstversorgung möglich; bei Amalgam ist dies zwar prinzipiell auch machbar, findet aber traditionell praktisch kaum Anwendung. Amalgam ist nach den gesetzlichen Vorgaben ein Medizinprodukt, das der Risikoklasse IIa zugeordnet und entsprechend zugelassen ist [4].

## Die öffentliche Diskussion um Amalgam

Obwohl Amalgam als verlässliches Restaurationsmaterial angesehen wird [5], steht es seit vielen Jahren in der – auch öffentlichen – Diskussion. Dabei wird hauptsächlich auf mögliche toxikologische Schäden durch das verwendete Quecksilber verwiesen [6–8]. Dies ist in Anbetracht des erheblichen Quecksilberanteils von Amalgam zunächst nicht abwegig, Mitte der 1990er-Jahre kam es aber zu teilweise sehr widersprüchlichen Bewertungen [9]. Nachdem die Amalgamdiskussion fast 2 Jahrzehnte auf einem konstant eher unaufgeregten Niveau geführt wurde, hat sie in jüngster Zeit vor allem durch die möglichen Auswirkungen des Quecksilbers auf die Umwelt neuen Auftrieb erhalten, insbesondere im Rahmen des Minamata-Übereinkommens, einem völkerrechtlichen Vertrag aus dem Jahr 2013 [10, 11], der eine weitgehende Vermeidung der Ausleitung anthropogenen Quecksilbers in die Umwelt zum Ziel hat. Nach ersten politischen Einschränkungen des Indikationsspektrums für Amalgam kam es nach der Ratifizierung des Minamata-Übereinkommens ab dem Jahr 2017 zu weiteren Limitationen.

Parallel zur kritischen Auseinandersetzung mit Amalgam hat sich die Adhäsivtechnik enorm weiterentwickelt, wodurch ein Paradigmenwechsel hin zu minimal-invasiven Restaurationen sowie deren effektiver Reparatur vollzogen wurde [12, 13]. Allerdings wurde auch bei diesen Werkstoffen die Frage nach der Verträglichkeit, z. B. durch freigesetztes Bisphenol A, das eine östrogenartige Wirkung hat, in der Öffentlichkeit diskutiert. Heute ist Kompositkunststoff das am häufigsten verwendete Material. Ebenso adhäsiv verankert werden Keramikinlays und -teilkronen, Letztere jedoch nur bei ausgedehnteren Sekundärdefekten und, bedingt durch die zahntechnische Herstellung, zu einem deutlich höheren Preis.

**Figure 1:**
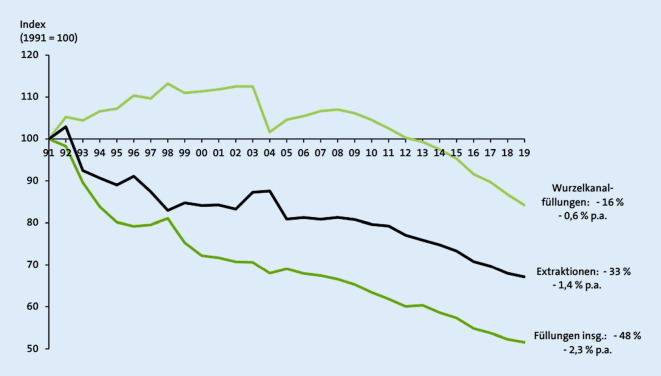

Ebenso beträchtlich wie der Materialsektor hat sich der „Faktor Patient“ weiterentwickelt. Auf der einen Seite ist die Mundgesundheit bezüglich der Karies seit den 1970er-Jahren deutlich besser geworden. In den letzten 30 Jahren konnte die Karies bei Jugendlichen um mehr als 80 % reduziert werden, wenn auch die Wurzelkaries bei Älteren deutlich zunimmt [14], und im gleichen Zeitraum nahm die Zahl der Füllungen um über 40 % ab ([15]; Abb. 1). Auf der anderen Seite tolerieren Patienten heute kaum noch nichtzahnfarbene Materialien, wobei hier zwischen Amalgam und Gold kein großer Unterschied in der (Nicht‑)Akzeptanz festzustellen ist – völlig entkoppelt von aller Evidenz zur Langlebigkeit.

## Amalgam – gestern und heute

**Figure 2:**
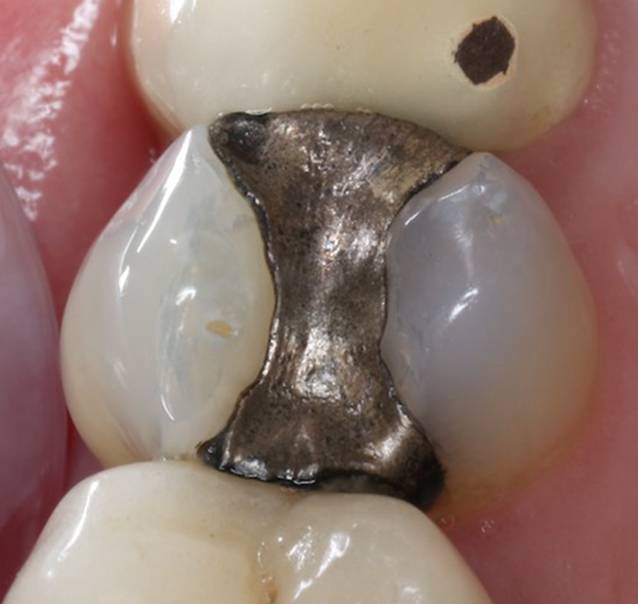

Der Begriff „Amalgam“ beschreibt kein spezifisches Material, sondern eine Werkstoffgruppe. Im Laufe der Zeit sind verschiedene Amalgame verwendet worden, die sich in Zusammensetzung und Eigenschaften deutlich unterscheiden. Bis in die Mitte des letzten Jahrhunderts wurde z. B. sog. Kupferamalgam verwendet, das im Vergleich zu den heute üblichen Amalgamen wesentlich mehr Quecksilber freisetzte und schlechtere Werkstoffeigenschaften aufwies. Heute werden silberbasierte Metallpulver für die sog. gamma-2-freien Amalgame verwendet, die eine vergleichsweise hohe Korrosionsresistenz aufweisen, wodurch die Freisetzung von Quecksilber reduziert wurde. Als Vorteile dieser Werkstoffe gelten die lange klinische Erfahrung, die vergleichsweise einfache Verarbeitung auch in tiefen, subgingivalen Bereichen (unterhalb des Zahnfleischsaums) aufgrund von Feuchtigkeitstoleranz und Schnitzbarkeit, die Langlebigkeit auch in ausgedehnten Kavitäten sowie die antibakteriellen Eigenschaften, die eine Sekundärkaries in vielen Fällen vermeiden helfen [16]. Klinische Nachteile des Amalgams sind seine Farbe (Abb. 2) und die Entfernung gesunder Zahnhartsubstanz, u. a. zum Erzeugen von Unterschnitten zur Retentionsgewinnung (Verbesserung des Halts der Restauration; [17]).

Die beschriebenen Faktoren haben im Verlauf der letzten 30 Jahre zu einer deutlichen Reduktion der Verwendung von Amalgam geführt. Heute bestehen nur noch 5,3 % der Füllungen im Rahmen der kassenzahnärztlichen Versorgung in Deutschland aus Amalgam [15]. In einem von der Europäischen Union (EU) bei der Firma Deloitte in Auftrag gegebenen Report (siehe auch unten) wurde eine Verringerung der Amalgamverwendung von 43 % in den letzten 10 Jahren ermittelt mit einer prognostizierten weiteren 12 %igen Reduktion pro Jahr. Wichtig ist auch ein Blick auf die Entwicklungen bei „Bestandsfüllungen“, bei denen man im Moment in Deutschland von 471 Mio. Füllungen ausgeht, die jedoch bis zum Jahr 2030 auf 407 Mio. zurückgehen sollen [18]. Im Jahr 1997 betrug der Anteil der Bestandsfüllungen mit Amalgam 58 % und im Jahr 2005 nur noch 43 %. Bei einer Trendfortschreibung dieser stark rückläufigen Entwicklung kann davon ausgegangen werden, dass der Anteil der bereits gelegten Füllungen mit Amalgam derzeit unter 30 % aller Bestandsfüllungen liegt.

Ein weiterer, für die Zukunft der Füllungstherapie entscheidender Faktor ist die Ausbildung an den Universitäten. Während bis vor 30 Jahren im Wesentlichen Amalgam und Gold für den Seitenzahnbereich gelehrt wurden, ist heute das Spektrum der verwendeten Biomaterialien wesentlich breiter und deutlich weniger „metallisch“. Seit vielen Jahren steht im Zentrum der universitätszahnmedizinischen Lehre die Prävention oraler Krankheiten, unter denen die Karies noch immer an vorderer Stelle steht. Zu diesem Konzept passt die defektorientierte Versorgung mit minimal-invasiven, unsichtbaren Kompositrestaurationen. Dies alles führt, wie auch oben erwähnt, zu einer Reduktion der Verwendung – einem natürlichen Phase Down von Amalgam.

## Amalgamalternativen

**Figure 3:**
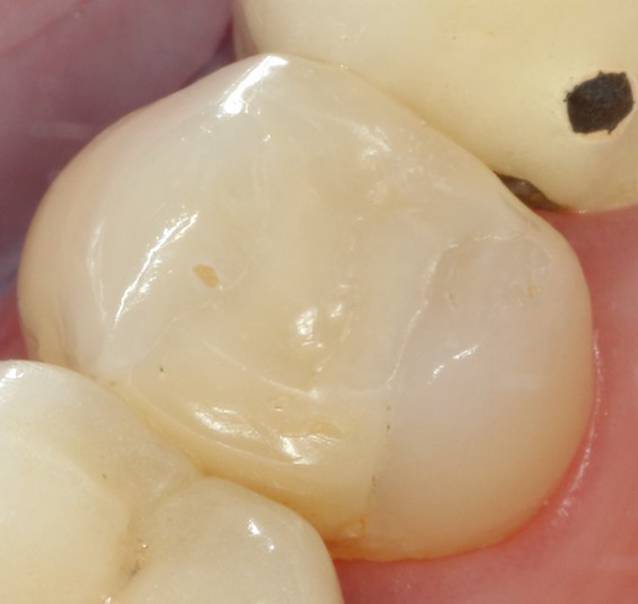

Da es sich beim Füllen von Zähnen um die Behandlung einer Krankheit handelt, müssen bei der Diskussion um das Amalgam auch die Eigenschaften möglicher alternativer Materialien erwogen werden. Hier sind zuerst die bereits erwähnten adhäsiv zu verarbeitenden dentalen Kompositkunststoffe („Füllungskunststoffe“) zu nennen (Abb. 3). Sie bestehen aus einer Kunststoffmatrix und anorganischen Füllern, daher der Name „Kompositkunststoff“. Die Matrix enthält sog. Monomere, d. h. organische Moleküle, die durch Energiezufuhr z. B. mittels eines speziellen Lichts aushärten. Als Monomere werden meist Bis-GMA (Bis-Glycidyldimethacrylat) und UDMA (Urethandimethacrylat) und Verdünner, wie z. B. TEGDMA (Triethylenglycoldimethacrylat), verwendet, die Füller bestehen aus unterschiedlichen Glasarten und Quarzen.

Heutige Kompositkunststoffe müssen aufgrund ihrer systemimmanenten Schrumpfung klebend (adhäsiv) am Zahn verankert, in Schichten eingebracht und separat lichtpolymerisiert werden. Dies bedeutet einen im Vergleich zum Amalgam deutlich gesteigerten Aufwand für den Behandler – u. a. auch bedingt durch besondere Maßnahmen der Trockenlegung. Neuere sog. Bulk-Fill-Kompositkunststoffe erlauben teilweise eine Insertion in dickeren Schichten (Inkrementen), der Aufwand für die minimal-invasive Präparation und für die besonderen Maßnahmen der Trockenlegung der Kavität bleibt jedoch derselbe [19–21].

Insgesamt stellen Kompositkunststoffe eine wissenschaftlich gut dokumentierte Amalgamalternative für bestimmte Indikationen dar [22], jedoch *keinen adäquaten Ersatz*. Im Vergleich zu Amalgamrestaurationen findet man bei solchen Kompositkunststoffen eine geringere Langlebigkeit insbesondere bei großen Defekten und schwierigen klinischen Situationen wie schlechter Mundhygiene und bestimmten Allgemeinerkrankungen [23]. Es ist wichtig, darauf hinzuweisen, dass der beim Kompositkunststoff gerade in der minimal-invasiven Ausführung bei Primärläsionen zu beobachtende Mehraufwand nicht nur in der spezifischen Materialapplikation begründet ist, sondern dass vor allem die z. T. unterminierende Kariesexkavation und defektorientierte kleine Präparation länger dauert als die meist ausgedehntere Amalgampräparation.

Eine Sonderform der Kompositkunststoffe sind die sogenannten Kompomere (aus *Kompo*sit und Glasiono*mer*), die besser als *polyacrylsäuremodifizierte Kompositkunststoffe* bezeichnet werden. Diese haben vor allem in der Kinderzahnheilkunde durch eine Reihe guter klinischer Daten große Bedeutung erlangt [24–26].

Ebenfalls als Amalgamalternativen werden Zemente diskutiert, v. a. die sogenannten *Glasionomeremente* (GIZ). Sie weisen eine chemische Haftung an den Zahnhartsubstanzen und eine (anfängliche) erhebliche Fluoridfreisetzung auf und sind relativ schnell zu verarbeiten. Leider sind GIZ neben ihren vielversprechenden biologischen Eigenschaften mechanisch grenzwertig stabil (Biegefestigkeit über 50 % geringer als bei Kompositkunststoffen). Daher erreichen sie gerade bei größeren Läsionen im kaulasttragenden Seitenzahnbereich bislang maximal die Indikation eines Langzeitprovisoriums [27, 28], obwohl es auch neuere klinische Daten gibt, die zumindest Hoffnung machen [29]. Bei kunststoffmodifiziertem GIZ konnte die Biegefestigkeit deutlich verbessert werden, allerdings auf Kosten der Abrasionsstabilität [30, 31]. Insgesamt betrachtet stellt die GIZ-Materialgruppe eine für die Zukunft interessante Linie dar.

Noch recht neu auf dem Markt sind *selbstadhäsive Komposithybride*. Hierbei handelt es sich um glasionomerähnliche, selbstadhäsive Kompositkunststoffe, die das Handling eines GIZ mit den (annähernden) werkstoffkundlichen Daten eines Kompositkunststoffs verbinden sollen. Die präklinische Evaluation verlief bereits erfolgreich [32, 33], klinische Daten fehlen jedoch weitgehend. Trotzdem stellt diese Materialgruppe im Moment einen interessanten Ansatz für einen *echten Amalgamersatz* dar.

Eine ebenfalls zu nennende Amalgamalternative sind *indirekte Restaurationen*, also Inlays und Teilkronen aus Gold, Keramik oder Hybridmaterialien [34, 35]. Diese sind klinisch in allen Belangen positiv evaluiert, aber aufgrund des beträchtlich höheren Preises (durch die technische Herstellung oder CAD/CAM-Prozesse) kein ebenbürtiger Ersatz für das plastische, günstige Amalgam.

Zusammenfassend kann festgehalten werden, dass in den letzten Jahrzehnten sowohl seitens der Industrie als auch der Universitäten erheblich Anstrengungen unternommen werden, einen Ersatz für das Amalgam zu finden. Dabei wurden auch durchaus Fortschritte erzielt, wobei jedoch in der Regel ein wesentlich erhöhter Behandlungsaufwand erforderlich wurde und bislang in schwierigen klinischen Situationen keine dem Amalgam ähnlichen Langzeiterfolge erzielt werden konnten.

## Verträglichkeit von Amalgam

Dieses Thema wurde in den vergangenen Jahrzehnten nicht nur von einer Vielzahl von Wissenschaftlern untersucht, sondern auch von nationalen und internationalen wissenschaftlichen Gremien wiederholt und eingehend analysiert. Kein anderes Füllungsmaterial wurde derart sorgfältig toxikologisch bewertet wie Amalgam. Beispielhaft soll hier der 2015 erschienene Bericht des „Scientific Committee on Emerging and Newly Identified Health Risks (SCENIHR)“ der Europäischen Kommission zur „Sicherheit von dentalem Amalgam und alternativer Materialien zur Zahnrestauration für Patienten und Benutzer“ [36] erwähnt werden.

Die Schlussfolgerung dieses sehr umfangreichen Berichtes war, dass lokale Nebenwirkungen in der Mundhöhle gelegentlich auftreten, meist in Form von lichenoiden (weißlichen) Schleimhautreaktionen in direkter Nachbarschaft von Amalgamfüllungen. Auch allergische Reaktionen auf Amalgam werden beschrieben. Die Häufigkeit dieser Nebenwirkungen ist insgesamt mit < 0,3 % für alle dentalen Werkstoffe jedoch gering. Für systemische Wirkungen, wie z. B. Schäden des zentralen Nervensystems, ist die wissenschaftliche Evidenz schwach. Auch alternative Materialien haben klinische Limitationen und bergen mögliche toxikologische Gefahren; hier sei beispielhaft auf die (wie auch bei Hg sehr geringe) Freisetzung des endokrinen Disruptors Bisphenol‑A (BPA) erwähnt. In einer kürzlich erschienenen Gegenüberstellung der Verträglichkeit von Amalgam und Kompositkunststoffen wurde gezeigt, dass aus beiden Materialgruppen biologisch aktive Substanzen freigesetzt werden, aber hinsichtlich der Verträglichkeit keine materialspezifische Reihung möglich ist [37]. Bei vorliegenden schweren Nierenschäden sollte kein Amalgam verwendet werden, da die Ausscheidung von Quecksilber eingeschränkt ist; Gleiches gilt im Übrigen auch für eine Reihe von Medikamenten, wie z. B. dem Antidiabetikum Metformin. Bei Schwangeren, so heißt es im SCENIHR-Report, ist wie bei jeder anderen medizinischen oder pharmazeutischen Intervention besondere Vorsicht bei jedweder zahnärztlichen Versorgung geboten. Es wird empfohlen, generell Zurückhaltung bei einer umfangreichen zahnärztlichen Therapie zu üben [38]. SCENIHR empfiehlt, auf die Verwendung von Amalgam bei Milchzähen im Allgemeinen zu verzichten, dies nicht wegen möglicher toxischer Schäden, sondern weil Milchzähne eine begrenzte Verweildauer im Mund haben und damit der Vorteil von Amalgam, nämlich eine höhere Langlebigkeit, nicht zum Tragen kommt. Andererseits kann hier ohne Schäden für die Patienten die Verwendung von Amalgam reduziert werden, was wiederum im Einklang mit dem Minamata-Übereinkommen (siehe unten) steht.

Zusammenfassend folgert SCENIHR, dass, hinsichtlich der Verträglichkeit auf der Basis vorliegender Evidenz, Amalgam oder alternative Materialien für die Allgemeinbevölkerung verwendet werden können. Die Wahl des Materials sollte jedoch die besondere Situation berücksichtigen bei Milchzähnen, Schwangerschaft, Allergien gegenüber Quecksilber oder anderen Bestandteilen von Füllungsmaterialien oder einer beeinträchtigten renalen Filtration bei schweren Nierenerkrankungen [36, 38].

Im Einklang mit diesen Ausführungen wird in einer im Jahr 2020 erschienen Stellungnahme der International Association for Dental Research (IADR) aus rein toxikologisch-wissenschaftlicher Sicht die Sicherheit von dentalem Amalgam für die allgemeine Bevölkerung ohne Allergien gegen Amalgambestandteile oder ohne schwere Nierenerkrankungen bestätigt [39].

## Quecksilber, Amalgam und die Umwelt

Bei der Diskussion um die Umweltbelastung durch Quecksilber steht die *atmosphärische Freisetzung* von Quecksilber – 2220 t Hg im Jahr 2015 [40] – im Vordergrund, wobei fossile Brennstoffe (ca. 24 %) und die Zement- oder Metallproduktion (28 %) die größte Rolle spielen. Hinsichtlich der geografischen Verteilung der globalen atmosphärischen Quecksilberemissionen stammen fast 40 % aus Ost- und Südostasien, während die EU (2015) mit 3,5 % eher wenig zu globalen atmosphärischen Quecksilberemissionen beiträgt [41].

Hauptquelle für die Einleitung von Quecksilber in *Boden/Grundwasser* ist der „handwerkliche und kleinteilige Goldabbau“ mit rund 1220 t Quecksilber (2015; [41]). Hinzu kommen 580 t (2015) aus anderen Quellen, zumeist aus Abfallbeseitigung (43 %), Erzabbau (40 %) und aus dem Energiesektor (17 %). Die gesamte anthropogene Einleitung von Quecksilber in Boden und Grundwasser betrug 2015 somit weltweit 1800 t. Die Quecksilberemission für Deutschland wird mit knapp 8 t im Jahr 2016 angegeben [3]. Die natürliche Quecksilberemission [42] z. B. durch Vulkanaktivität betrug weltweit im Jahr 2010 ca. 825–1335 t.

In diesem Zusammenhang wird oft die *Verwendung* von Hg für Amalgam thematisiert, die im Jahr 2018 in der EU geschätzt 27–58 t betrug, übrigens 43 % weniger gegenüber 2010 [43, 44]. Dabei ist zu berücksichtigen, dass für die Umweltbelastung nicht die verwendete Menge von Amalgam von Bedeutung ist, sondern die Menge, welche in die Umwelt gelangt. Dies geschieht hauptsächlich beim Entfernen alter Amalgamfüllungen und beim Legen neuer. Demgegenüber spielen andere Quellen, wie z. B. die Freisetzung während der Kremation mit 25 kg (Deutschland) pro Jahr (im Vergleich zu Kohlekraftwerken mit 5000 kg), eine eher geringe Rolle [45, 46]. In Deutschland sind aufgrund strikter Vorgaben der Abwasserverordnung seit den 1990er-Jahren in Zahnarztpraxen flächendeckend Amalgamabscheider gemäß DIN EN ISO 11143 zu verwenden, durch die mindestens 95 % der Amalgampartikel aus dem Abwasser zurückgehalten werden müssen. Die Entsorgung der so aufgefangenen Amalgamreste erfolgt durch speziell zertifizierte Firmen und das Quecksilber wird danach recycelt. Bei der Berechnung der Umweltbelastung muss außerdem vor allem auf den Bestand an Amalgamfüllungen (ca. 30 % der vorhandenen Füllungen nach Angaben der Kassenzahnärztlichen Bundesvereinigung – KZBV) Bezug genommen werden, und nicht primär auf den Verbrauch. Nach Angaben der KZBV wurden 2019 knapp 50 Mio. plastische Füllungen gelegt, rechnet man Privatpatienten hinzu, so kommt man auf ca. 55 Mio. Füllungen. Dazu kommen ca. 11 Mio. Kronen (10 Mio. GKV, 1 Mio. privat), bei denen man auch annehmen kann, dass z. T. (siehe Bestandsanteil) alte Amalgamfüllungen entfernt wurden. Errechnet man nun die Ausleitung von Quecksilber über Amalgamabscheider bei einer 95 %igen Abscheidung und 50 % Füllungserneuerung ([47]; d. h., alte Füllung wurde entfernt), so ergibt sich für Deutschland eine geschätzte maximale Ausleitung von weniger als 0,5 t, d. h. ca. 8 g pro Zahnarzt jährlich. Da die Zahl der neuen Amalgamfüllungen mit 5,3 % heute schon wesentlich unter dem Bestand (30 %) liegt, ist davon auszugehen, dass diese Menge in Zukunft auch ohne jegliche Regelung weiter abnimmt.

Interessanterweise wurde das Thema Amalgam und Umwelt auch von der EU aufgegriffen: 2014 erschien ein Bericht „Scientific Committee on Health and Environmental Risks (SCHER)“ zu „Umweltrisiken und indirekten Gesundheitsfolgen von Quecksilber aus dentalem Amalgam“ [42]. In diesem Bericht werden 3 verschiedene Expositionsszenarien (hoch, durchschnittlich, gering) analysiert, je nachdem wie groß die Ausleitung von Amalgampartikeln in die Umwelt ist. Für die heutige Situation in Deutschland ist im Wesentlichen das zweite, eher das dritte Szenario von Bedeutung, bei denen – wie in Deutschland flächenhaft erfolgt – die Installation von Amalgamabscheidern vorliegt. SCHER kommt zu dem Schluss, dass nur bei der höchsten Exposition unter extremen lokalen Bedingungen (maximale Zahnarztdichte, maximaler Quecksilberverbrauch, Fehlen von Amalgamabscheidern) das Risiko einer Sekundärvergiftung durch Methylierung von freigesetztem Quecksilber nicht ausgeschlossen werden kann. Hinsichtlich des Risikos für die menschliche Gesundheit durch Quecksilber in Boden und Luft aus der Verwendung von dentalem Amalgam kann geschlossen werden, dass dieser Emissionsanteil von Hg einen sehr geringen Beitrag zur Gesamtexposition des Menschen aus dem Boden und durch Inhalation darstellt. Die verfügbaren Informationen zu den Hg-freien Alternativen lassen keine solide Risikobewertung für die Umwelt zu: Das Risiko wird zwar als gering eingeschätzt, aber es gebe keine wissenschaftlichen Daten, die dieses untermauern [42].

## Minamata-Übereinkommen und Amalgam

Bereits seit den 1970er-Jahren ist man im Rahmen des United Nations Environment Programme (UNEP) bestrebt, die anthropogene Umweltbelastung mit Quecksilber zu reduzieren. 2009 wurde von der UNEP beschlossen, einen weltweit gesetzlich bindenden Vertrag zur Reduktion der Quecksilberemission in die Umwelt zu initiieren. Nach 5 internationalen Konferenzen (2009 bis 2013) wurde 2013 ein Vertrag in der japanischen Stadt Minamata unterschrieben („Minamata-Übereinkommen“). In dieser Stadt hatten in den 50er-Jahren des letzten Jahrhunderts weit mehr als 2000 Menschen massive Schäden des Zentralnervensystems erlitten, die auf die Ausleitung von Methylquecksilber aus der chemischen Industrie in das Meer zurückgeführt werden konnten [10]. Mittlerweile ist das Minamata-Übereinkommen von 128 Staaten ratifiziert worden, auch von der EU, und es ist damit rechtsverbindlich für diese Staaten.

Wenn auch – wie oben dargestellt – der Beitrag der Zahnmedizin zur Quecksilberbelastung der Umwelt, hier vor allem der Atmosphäre, vergleichsweise gering ist, wurden Quecksilber enthaltende Produkte, wie z. B. Amalgam, bei der Verhandlung zum Minamata-Übereinkommen eingehend diskutiert. Im Gegensatz zu den meisten in diesem Übereinkommen aufgeführten Hg-haltigen Produkten, wird Amalgam zur Behandlung einer Erkrankung benötigt. Als Kompromiss zwischen Fragen der Umweltbelastung durch Quecksilber aus Amalgam einerseits und den Erfordernissen der medizinischen Versorgung der Patienten (Versorgungssicherheit) andererseits wurde einvernehmlich, d. h. auch mit ausdrücklicher Zustimmung der betroffenen Zahnärzteorganisation, wie z. B. der FDI World Dental Federation, eine Reduktion der Verwendung von Amalgam (Phase Down), jedoch ohne eine Zeitvorgabe beschlossen. Dabei wurde das Phase Down an eine Reihe von Maßnahmen geknüpft, von denen mindestens 2 erfüllt sein müssen. Diese betreffen hauptsächlich Forderungen nach verbesserter Prävention, vermehrter Forschung zu neuen Füllungswerkstoffen als Ersatz von Amalgam und umfangreichere Ausbildung über Hg-freie Füllungsmaterialien (Einzelheiten siehe [10]).

Im Minamata-Übereinkommen wurde auch festgelegt, dass in meist zweijährigem Abstand sog. Conferences of Parties (COP) durchgeführt werden. Dabei werden weitere Beschlüsse gefasst (siehe Minamata-Homepage http://mercuryconvention.org/). Die dritte Konferenz (COP 3) hat im November 2019 beschlossen, dass Unterlagen zu einem Phase Out von Amalgam eingereicht werden sollten, und darüber soll bei der nächsten Konferenz (COP 4) diskutiert werden, die im November 2021 (1. Teil) und im März 2022 (2. Teil) stattfindet.

## Regelungen in der Europäischen Union

Als Folge der Ratifizierung des Minamata-Übereinkommens durch die EU wurde 2017 eine neue Quecksilberverordnung [48] erlassen. Darin sind auch Bestimmungen zur Verwendung von Amalgam enthalten, die für alle EU-Staaten verbindlich sind (Artikel 10). Danach darf seit dem 01.01.2019 Dentalamalgam nur noch in vordosierter, verkapselter Form verwendet werden. Die Verwendung von Quecksilber in loser Form durch Zahnärzte ist verboten. Außerdem darf seit dem 01.07.2018 Dentalamalgam nicht mehr für die zahnärztliche Behandlung von Milchzähnen, von Kindern unter 15 Jahren und von Schwangeren oder Stillenden verwendet werden, es sei denn, der Zahnarzt erachtet eine solche Behandlung wegen der spezifischen medizinischen Erfordernisse bei dem jeweiligen Patienten als zwingend notwendig. Amalgamabscheider nach ISO 11143 sind zu installieren und die Entsorgung der Amalgamreste muss durch lizensierte Unternehmen erfolgen. Zudem legt jeder Mitgliedstaat zum 01.07.2019 einen nationalen Plan mit den Maßnahmen vor, die er zu ergreifen beabsichtigt, um die Verwendung von Dentalamalgam schrittweise zu verringern.

Außerdem hatte die EU-Kommission (Generaldirektion Umwelt) bis zum 30.06.2020 dem Europäischen Parlament und dem Rat einen Bericht über das Ergebnis ihrer Bewertung vorzulegen, ob es möglich sei, die Verwendung von Dentalamalgam auf lange Sicht und vorzugsweise bis 2030 schrittweise auslaufen zu lassen (sog. Phase Out), wobei den nationalen Plänen (s. oben) Rechnung getragen und die Zuständigkeit der Mitgliedstaaten in den Bereichen Organisation des Gesundheitswesens und medizinische Versorgung uneingeschränkt geachtet werden musste. In diesem Zusammenhang hatte die EU-Kommission die Firma Deloitte beauftragt, ein entsprechendes Gutachten zu erstellen. In diesem Gutachten [43] und der darauf fußenden Stellungnahme der EU-Kommission (Generaldirektion Umwelt; [49]) wird ein Phase Out der Verwendung von Amalgam als „technisch und ökonomisch vor 2030 durchführbar“ bezeichnet. Die Generaldirektion Umwelt will dem Europäischen Parlament und dem Rat 2022 ein entsprechendes Gesetz vorschlagen.

Sowohl der Deloitte-Report als auch der darauf basierende Vorschlag der Generaldirektion Umwelt wurden vom „Council of European Dentists“ eingehend analysiert [43]. Dabei ergab sich zusammenfassend, dass die beiden von der Europäischen Kommission vorgelegten Berichte zunächst das ihnen erteilte Mandat, die besonderen Bedürfnisse der nationalen Gesundheitssysteme zu berücksichtigen, nicht beachtet haben. Sie vernachlässigen die wirtschaftlichen Folgen für die nationalen Gesundheitssysteme und die damit verbundene Gesundheit der Patienten, insbesondere jetzt, da die COVID-19-Pandemie die Gesundheitslandschaft erheblich verändert hat. In Deutschland wird nach Angaben der Bundesregierung bei einem Phase Out mit ca. 1 Mrd. Euro jährlicher Mehrkosten für die Kostenträger gerechnet [3]. Außerdem ist die wissenschaftliche Grundlage für die Schlussfolgerungen dieser Berichte (Deloitte-Report und Bericht Generaldirektion Umwelt) schwach, da mehr als 50 % der erforderlichen Daten aus EU-Ländern fehlen. Der Bedarf an vorbeugenden Maßnahmen und mehr Forschung zu Ersatzmaterialien wird im Deloitte-Report angesprochen, jedoch in dem Bericht der EU-Kommission nicht ausdrücklich erwähnt. Die Generaldirektion hat diese kritischen Anmerkungen zur Kenntnis genommen und zu einer weiteren Konsultation im Laufe des Jahres 2021 eingeladen. Von besonderer Bedeutung ist dabei, dass die Zuständigkeit für die Sozialsysteme bei den Mitgliedsstaaten liegt [50] und dass darauf in beiden Dokumenten keine Rücksicht genommen wurde. Bislang wurde auch die Generaldirektion „Gesundheit“ nicht in die Diskussion eingebunden.

Sowohl die Weltgesundheitsorganisation (WHO; [51]) als auch die FDI [52] als Sprecher aller nationalen Zahnärzteorganisationen unterstützen ausdrücklich einen Phase Down. Ein Phase Out (quasi eine Nichtverfügbarkeit von Amalgam) wird aber als nicht sachdienlich abgelehnt, da die zahnärztliche Versorgung der Patienten (Versorgungssicherheit) wesentlich beeinträchtigt wird. Dies wird insbesondere sogenannte vulnerable Gruppen wie Pflegebedürftige und Patienten mit Behinderungen treffen, die z. B. aus medizinischen Gründen keine hinreichende Zahnpflege betreiben können und bei denen Kompositfüllungen eine wesentlich geringere Lebensdauer haben. Gleiches gilt für Patienten mit z. B. durch Alter und Medikamente bedingtem stark reduzierten Speichelfluss. Hinzu kommen die höheren Kosten für Restaurationen mit alternativen Werkstoffen (siehe oben). Dies kann zu einer Zunahme von Extraktionen und damit zu einer erhöhten sozialen Ungleichheit (Inequality) führen, insbesondere bei Menschen aus sozioökonomisch schwachen Gebieten, auch weil diese zusätzlich unter einem erhöhten Kariesaufkommen leiden [53]. Erfahrungen aus Norwegen nach dem dort verhängten Phase Out zeigten, dass Füllungen mit Amalgamalternativen zeitaufwendiger, teurer und weniger langlebig waren [54, 55].

Interessanterweise wurde im Deloitte-Report selbst erwähnt, dass in den letzten 10 Jahren die Amalgamverwendung um ca. 43 % zurückgegangen ist und dass in Zukunft mit einer weiteren jährlichen Reduktion von ca. 12 % pro Jahr gerechnet wird („natürliches Phase Down“). Auf Deutschland projiziert hieße das, dass 2030 noch 1 % Amalgam als Füllungsmaterial verwendet wird – ohne irgendeine gesetzliche Regelung (d. h. ohne Phase Out). Hinzu kommt, dass – wie oben ausgeführt [42] – für die Umweltbelastung eine derart geringe Amalgamverwendung keine Rolle spielt, sondern eher die Entfernung bestehender Amalgamfüllungen. Selbst im Deloitte-Report wird darauf hingewiesen, dass ein Phase Out keine signifikante Auswirkung auf die Quecksilberausleitung bis 2030 hat. Leider wurde das weder in der Schlussfolgerung des Deloitte-Reports noch in der Stellungnahme der Generaldirektion Umwelt aufgegriffen. Man muss daher davon ausgehen, dass hier eine weitgehend politische Entscheidung vonseiten der Generaldirektion Umwelt getroffen wurde.

## Regelungen in Deutschland

**Figure 4:**
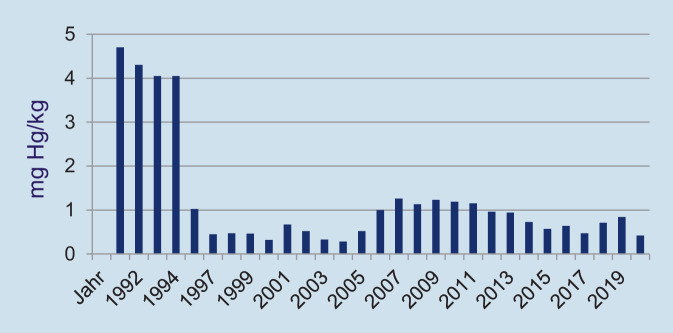

Schon 1997 haben das Bundesministerium für Gesundheit (BMG) und das Bundesinstitut für Arzneimittel und Medizinprodukte (BfArM) zusammen mit verschiedenen zahnärztlichen Organisationen ein Konsenspapier „Restaurationsmaterialien in der Zahnheilkunde“ veröffentlicht, in dem Zahnärztinnen und Zahnärzten empfohlen wird, bei Schwangeren unabhängig vom Füllungsmaterial mit umfangreichen Füllungstherapien bis nach der Geburt ihres Kindes zu warten und bei der Behandlung von Kindern sorgfältig zu prüfen, ob eine Amalgamtherapie notwendig ist [3]. Zudem wurden bereits, wie oben dargestellt, in den 1990er-Jahren – und damit weit vor den von der EU erlassenen Vorgaben – in Deutschland flächendeckend Amalgamabscheider gemäß ISO 11143 installiert [3]. Die dadurch erfolgte deutliche Reduktion der Quecksilberemission in die Umwelt kann beispielhaft an Daten des Klärwerkes Regensburg (Abb. 4) veranschaulicht werden.

Die oben erwähnten EU-Regelungen, wie z. B. die Medizinprodukterichtlinie (gültig bis Mai 2021), die EU-Medizinprodukte-Verordnung (gültig ab Mai 2021) sowie die EU-Verordnung zur Reduktion von Quecksilber (2017), sind unmittelbar geltendes nationales Recht bzw. in solches Recht überführt worden. Vorgaben der EU-Verordnung zur Reduktion von Quecksilber in der Umwelt sind hinsichtlich der Verwendung von Amalgam durch die Kassenzahnärztliche Bundesvereinigung und den GKV-Spitzenverband fristgerecht umgesetzt worden (zu den Einzelheiten siehe [38]).

## Ausblick

Die Kontroverse um das Amalgam mutet wie eine unendliche Geschichte an, bei der auf der einen Seite die Vorteile bei der Behandlung einer Krankheit (meist Zahnkaries), vor allem komplizierter Zahndefekte unter schwierigen klinischen Situationen, stehen. Andererseits wird eine mögliche Toxizität postuliert und in den letzten Jahren auch die Belastung der Umwelt. Die Patienten bevorzugen aus ästhetischen Gründen vermehrt zahnfarbene Alternativen (meist Kompositkunststoffe). Die Abnahme der Verwendung von Amalgam wird sich auch ohne zusätzliche gesetzliche Reglungen in Zukunft fortsetzen (natürlicher Phase Down). Probleme der alternativen Werkstoffe sind aber immer noch der erhöhte Aufwand und die damit verbundenen höheren Kosten sowie eine verringerte Langlebigkeit, die sich besonders in schwierigen Fällen und bei vulnerablen Gruppen ungünstig auswirkt. Interessanterweise entfaltet die Politik auf diesem Gebiet eine erstaunliche Regelungsaktivität, wohl auch als Reaktion auf öffentlichkeitswirksame Aktionen von entsprechenden Interessengruppen. Diese z. T. politisch motivierten Regelungen findet man in anderen durchaus vergleichbaren Bereichen, wie zum Beispiel bei der Verordnung von Arzneimitteln, nicht in diesem Maß.

Charakteristisch für diese Diskussion ist, dass sie größtenteils materialorientiert geführt wird: Amalgam – Ja oder Nein. Dabei hat sich in vielen Untersuchungen gezeigt, dass die Situation beim individuellen Patienten eine entscheidendere Rolle spielt [23], wie z. B. die Größe des Defektes, die Mundhygiene, aber auch Allgemeinerkrankungen. Die Frage sollte also vielmehr lauten: In welchen Fällen welches Material? Wegen der vielen, einer strikten Regelung nicht zugänglichen individuellen Situationen jedes einzelnen Patienten ist es vielmehr sinnvoller, eine Zahl von Materialien verfügbar zu haben, aus der dann im Einzelfall – zusammen mit dem Patienten – das jeweils geeignete Material ausgewählt werden kann. Auch die Bundesregierung hat in ihrem Nationalen Aktionsplan [15] entsprechend deutlich gemacht, dass aus ihrer Sicht der Einsatz von Amalgam weiter möglich bleiben soll. Diese Entwicklung hat sich in Deutschland in den vergangenen mehr als 20 Jahren bewährt und sollte nicht ohne Grund, d. h. ohne verlässlichen Amalgamersatz, verlassen werden. Diverse Forschungsbemühungen in dieser Zeit haben zwar innovative Materialien hervorgebracht, die jedoch schließlich keinen klinischen Vorteil brachten. Neue Materialien im Sinne eines echten Amalgamersatzes zu entwickeln, muss daher oberstes Ziel gemeinsamer Anstrengungen von Wissenschaftlern aus Zahnmedizin, Naturwissenschaften und Industrie sein und durch entsprechende Mittel z. B. auch seitens der Europäischen Union gefördert werden.
